# Evaluation of Therapeutic Opioids in Hair of Neonatal and Pediatric Patients

**DOI:** 10.1002/dta.3935

**Published:** 2025-07-27

**Authors:** Max Polke, Florian Zapf, Tanja Restin, Thomas Kraemer, Tina M. Binz

**Affiliations:** ^1^ Center for Forensic Hair Analysis, Zurich Institute of Forensic Medicine University of Zurich Zurich Switzerland; ^2^ Department for Pediatric Intensive Care Medicine and Neonatology University Children's Hospital Zurich Zurich Switzerland; ^3^ Department of Neonatology University Hospital and University of Zurich Zurich Switzerland; ^4^ Department of Forensic Pharmacology and Toxicology, Zurich Institute of Forensic Medicine University of Zurich Zurich Switzerland

**Keywords:** children, fentanyl, hair analysis, LC–MS/MS, opioids

## Abstract

Forensic hair analysis poses a valuable tool for assessing opioid exposure in children and neonates. However, reliable literature data on opioid concentrations in the hair of this population are mostly scarce, making the interpretation of such hair analysis results rather challenging. This noninterventional study aims to address this issue by investigating 118 hair samples of pediatric patients (median age: 50 days) from the University Children's Hospital Zurich. These patients were treated with medically approved novel synthetic opioids (fentanyl, remifentanil, sufentanil, or alfentanil) and traditional opioids (morphine, methadone, and hydromorphone) during their clinical treatment. Quantification of the opioids and selected metabolites was achieved by a previously validated liquid chromatography–tandem mass spectrometry (LC–MS/MS) based method, which showed good sensitivity with lower limits of quantification (LLOQs) ranging from 0.1 to 1 pg/mg hair. Most analytes were successfully detected in patients' hair, with the majority being identified for the first time in this matrix. Significant correlations were found between the opioid concentrations in hair and the administered medication doses, indicating that hair analysis may reflect the extent of opioid exposure in this population. Furthermore, metabolite ratios similar to the ones commonly found in adult hair were identified, which are forensically important to differentiate between active intake of a drug and contamination. The metabolite ratio of β‐hydroxyfentanyl to fentanyl was particularly well suited for children and neonatal patients. In conclusion, concentration ranges, metabolite ratios, and dose correlations of the studied opioids in pediatric hair were established, providing insights into opioid incorporation pathways.

## Graphical abstract

This study investigated the incorporation of novel and traditional medically used opioids into the hair of 118 pediatric and neonatal patients. Concentrations (median [range]) in hair were as follows: fentanyl: 4.03 pg/mg [0.10–343], sufentanil: 0.31 pg/mg [0.10–8.64], remifentanil: 0.14 pg/mg [0.10–0.43], and morphine: 27.4 pg/mg [1.0–1870]. The results of this study will contribute to the interpretation of hair testing results in children and newborns and therefore play an important role in forensic toxicology.

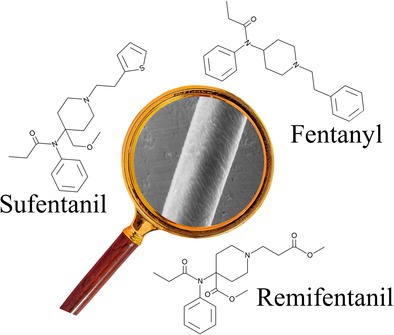

## 1 Introduction

There is arguably no other class of substances with such a dual role as opioids. In a medical context on the one hand, opioids are indispensable for pain management during major surgery and in palliative care, where they effectively relieve acute and chronic pain [1]. On the other hand, opioids (newer synthetic opioids in particular) pose a substantial risk to public health due to their high addiction potential and acute toxicity [2]. The destructive effects of opioids extend far beyond the drug‐consuming individual and have a profound impact on communal and social structures, particularly in family environments. Forensic hair analysis is a valuable tool to evaluate the prevalence of such opioids at both individual and societal levels [3, 4]. In contrast to more traditional matrices such as blood or urine, hair offers a significantly extended detection window, allowing the detection of most analytes and their metabolites after months or even years [5]. In addition, hair sampling is completely noninvasive, making it highly suitable for vulnerable populations such as children and neonates who can get exposed to opioids through various routes [6].

Interpreting hair analysis results is, however, particularly challenging in children and neonates due to their porous hair structure, which facilitates radial diffusion of substances from external sources such as sweat, sebum, or environmental contamination [7]. Reliable reference data from literature is essential to counteract the above‐mentioned difficulties. However, such data for opioids in the hair of children and neonates is scarce since clinical studies are difficult to realize due to the harmful nature of the substance class. Thus, the currently available data is predominantly based on isolated case reports with numerous unknown factors, including self‐reported opioid consumption—which is known to be often inaccurate [8]. The lack of normative concentration ranges and knowledge on dose–response relationships, for example, limits statements about the extent or duration of exposure to a particular opioid when interpreting hair analysis results. In fact, some research teams such as Alvarez et al. even suggested that hair analysis does not allow discrimination between acute and chronic exposure to drugs in young children at all [9]. Furthermore, the lack of reference data also concerns the proportional relationship between the major metabolites and the parent drugs in hair. Reference metabolite ratios can allow differentiation between active intake and contamination, which is of utmost importance for the interpretation of hair analysis results [10].

This study aims to address the above‐described lack of reliable literature data on opioids and metabolites in the hair of children and neonates. To this end, we investigated authentic hair samples from young patients who received opioids (fentanyl, sufentanil, remifentanil, alfentanil, morphine, hydromorphone, and methadone) under strictly monitored clinical conditions. The primary objective was to quantify these opioids in hair using a previously developed and validated LC–MS/MS‐based hair analysis method. Thereby, we aimed to establish opioid‐specific concentration ranges in patients' hair and determine possible correlations with the administered opioid dose and the duration of administration. Additionally, selected metabolites should be quantified and their respective ratios to the parent drugs should be assessed. For the parent drug fentanyl, three metabolites were evaluated: norfentanyl, β‐hydroxyfentanyl, and 4‐anilino‐*N*‐phenethylpiperidine (4‐ANPP). For the other opioids, the metabolites norsufentanil (parent drug sufentanil), remifentanil‐acid (parent drug remifentanil), and hydromorphone (parent drug morphine) were evaluated. Subordinate objectives included comparing the results obtained with existing literature and in‐house data from routine case work, where available.

## 2 Material and Methods

### 2.1 Study Design

This single‐center study with a noninterventional prospective study design was conducted in collaboration with the University Children's Hospital (Zurich, Switzerland) and the Zurich Institute of Forensic Medicine, Center for Forensic Hair Analysis (Zurich, Switzerland). It was approved by the Swiss Ethics Board (approval number: 2022‐01693/amendment approval date: 09.01.2024) and registered at ClinicalTrial.gov (Identifier NCT05740657, released on 13.02.2023).

### 2.2 Study Inclusion and Data Collection

Patient recruitment took place at the Pediatric Intensive Care Unit (PICU) of the main study site (University Children's Hospital Zurich). All patients admitted to the PICU were constantly assessed for study eligibility by the local study investigator during daily clinical routine. Study inclusion was possible for newborns, infants, children, and adolescents up to 13 years of age that received opioids during their hospitalization, for example, during surgery or as part of post‐operative pain management. The medication regimen had to include fentanyl or fentalogs (sufentanil, remifentanil, or alfentanil) and optionally one or more traditional opioids such as morphine, hydromorphone, and methadone. The last dose of opioids had to be administered no more than 3 months prior to study enrollment. Furthermore, only patients with a sufficient amount of head hair and without any cosmetic hair treatment like coloring or bleaching were considered for inclusion. Upon fulfillment of all the study inclusion criteria, the nature of the study was explained to the patients and/or legal representatives (usually the parents).

By signing the informed consent form, patients and/or legal representatives agreed to the planned interventions (hair sampling) and the collection of specific patient and medication‐related data in a pseudonymized form. More specifically, this information included: the patient ID, full name, date of birth, weight, gender, and hair color. Medication‐related data included: the type and total cumulative amount (μg per kilogram body weight [μg/kgBW]) of opioid administered, duration of medication, date of last opioid administration, and potential intrauterine exposure of mothers to opioids during birth or C‐section.

### 2.3 Pediatric Hair Sampling and Sample Preparation

Hair samples were taken by the local study investigator at the PICU at the University Children's Hospital Zurich. The sampling followed the established protocol of the Center for Forensic Hair Analysis [11]. Due to the limited amount of head hair and the generally finer hair diameter in young children and neonates, it was not always feasible to tie the hair samples. In such instances, hair collection was performed nonetheless, with the hair gathered as a loose bundle. The hair sample was cut as close to the scalp as possible, ensuring that the remaining hair length on the head did not exceed 1–2 mm. The collected hair was subsequently wrapped in aluminum foil within a labeled sampling kit and transported to the Center for Forensic Hair Analysis. Prior to further sample preparation, the exact weight and hair color were documented for every hair sample.

Hair sample preparation and extraction followed a so‐called one‐pot approach, where pulverization and extraction are performed in the same vessel (Eppendorf tube) to minimize the risk of external contamination and especially sample loss [12]. This procedure is efficient for the extraction of various substance classes, including synthetic and semisynthetic opioids, and was considered ideal for the small hair sample quantities in this study. Prior to extraction, the routinely used protocol at the Center for Forensic Hair Analytics normally includes a successive washing step with water, acetone, and hexane, mainly to remove external contaminations [12, 13]. This step was omitted in the present study due to the extremely small hair quantities (< 2 mg on average), as washing would have resulted in excessive sample loss.

### 2.4 Reference Substances and Chemicals

Methanol or acetonitrile standard solutions (1 mg/mL) of 4‐ANPP, acetylcodeine, 6‐monoacetylmorphine, alfentanil, dihydrocodeine, fentanyl, hydrocodone, hydromorphone, methadone, morphine, and pethidine were purchased from Cerilliant (Round Rock, USA). The reference standards codeine, naloxone, norfentanyl, oxycodone, oxymorphone, tramadol, as well as the deuterated standards (0.1 mg/mL) fentanyl‐d_5_, methadone‐d_9_, and morphine‐d_3_ were purchased from Lipomed (Arlesheim, Switzerland). Methanolic solutions (1 mg/mL) of norsufentanil, remifentanil, remifentanil‐acid, and sufentanil, and 0.1 mg/mL solutions of the deuterated standards norfentanyl‐d_5_ and norsufentanil‐d_3_ were purchased from Cayman Chemical (Ann Arbor, USA). LGC (Wesel, Germany) supplied the β‐hydroxyfentanyl (1 mg/mL) standard. LC–MS grade methanol (Chromasolv), purchased from Sigma‐Aldrich (Buchs SG, Switzerland) was used for the preparation of the working standard solutions. LC–MS grade acetonitrile, purchased from ACROS ORGANICS (Fisher Scientific AG, Switzerland), LC–MS grade water (Chromasolv) from Sigma‐Aldrich, and ammonium formate and formic acid, obtained from Merck (Darmstadt, Germany), were used for the LC–MS/MS mobile phases.

### 2.5 Preparation of Calibrators and Quality Control Samples

During sensitivity experiments, the limit of detection (LOD) and lower limit of quantification (LLOQ) values for the different analytes were evaluated by measuring calibrations in the low concentration range (below 2 pg/mg hair). Based on their LLOQ value, the analytes were assigned to one of two groups (Table S1). For each group, three calibrator solutions were then prepared at nominal concentrations of 0.2, 2, and 200 ng/mL for group one and 2, 200, and 2000 ng/mL for group two. Furthermore, an internal standard (IS) solution containing fentanyl‐d_5_, norfentanyl‐d_5_, norsufentanil‐d_3_, methadone‐d_9_, and morphine‐d_3_ each at a concentration of 40 pg/μL in methanol was prepared. Both the internal standard and calibrator solutions were stored at −20°C in amber vials until further use. QCs (QC_low_, QC_med_, and QC_high_) and calibrator samples were freshly prepared before each run by spiking ~20 mg opioid‐free scalp hair with the calibrator solutions and 50 μL IS mix. The resulting calibration ranges were 0.1 to 500 pg/mg for group 1 and 1 to 5000 pg/mg for group 2. Details on the QC and calibrator concentrations in hair can be obtained from Table S2. The opioid‐free scalp hair originated from drug‐abstinent volunteers at the laboratory of the Center for Forensic Hair Analytics in Zurich, Switzerland, who had given oral consent. The scalp hair was tested for absence of all common opioids using the multi‐analyte approach by Scholz et al. [12].

### 2.6 Instrumentation

A Prominence UFLC system (Shimadzu, Kyoto, Japan) equipped with a Kinetex F5 column (100 mm × 2.1 mm, 100 Å, 2.6 μm, Phenomenex) coupled with an ultra‐high performance liquid chromatography (UHPLC) SecurityGuard ULTRA Cartridge F5 (2.1 mm ID) was employed in this study. The column and the autosampler were maintained at 40°C and 15°C, respectively. The binary mobile phase consisted of A: water with 1 mM ammonium formate and 0.1% formic acid and B: acetonitrile with 1 mM ammonium formate and 0.1% formic acid. The sample injection volume was 5 μL and the subsequent gradient elution was at a flow rate of 0.6 mL/min using the following time program: 0–1.5 min maintaining eluent B at 3%; 1.5–9 min increasing to 60% eluent B; 9–10 min increasing to 95% eluent B; 10–11 min maintaining eluent B at 95%; 11.01 min returning to starting conditions (3% eluent B) and maintaining the gradient until the end of the run at 13 min.

For analyte detection, a QTRAP 7500 from Sciex (Darmstadt, Germany) was operated in multiple reaction monitoring (MRM) mode, utilizing positive electrospray ionization at 1500 V. Nitrogen was used as the curtain gas (fixed at 42 psi) and the source (OptiFlow Pro) was maintained at a temperature of 500°C. For identification and quantification, two individually optimized MRM transitions were used for each analyte, applying a detection window of ±15 s around the respective elution time. This “scheduled MRM” mode enables higher selectivity and sensitivity. The retention times and MS parameters can be obtained from Table S3.

### 2.7 Method Validation

The analytical validation experiments performed in this work were based on the guidelines of the Society of Hair Testing (SoHT) and the Society of Toxicological and Forensic Chemistry (GTFCh) [14, 15, 16]. Validation took place in terms of selectivity, specificity (including matrix effects), sensitivity (limit of detection (LOD) and lower limit of quantification (LLOQ)), linearity, accuracy, precision (intraday and interday) and recovery (extraction efficiency). Post‐sample preparation, the hair sample extracts from this study were immediately transferred to the LC–MS/MS system and analyzed. Major analyte stability concerns due to factors such as freezing and thawing of sample extracts were therefore not expected; thus, no sample stability experiments were performed as part of the validation of this work.

### 2.8 Statistical Analysis

Statistical analyses were conducted using Prism 10 (GraphPad Software, CA, USA). Prior to correlation analysis between hair concentrations of opioids and their metabolites and the corresponding administered doses, data were assessed for normality using the Shapiro–Wilk test. As the data did not follow a normal distribution, nonparametric Spearman's rank correlation coefficients were calculated. *p*‐values > 0.05 were considered as not statistically significant (ns); *p* < 0.05 as significant; *p* < 0.01 as very significant; *p* < 0.001 and *p* < 0.0001 as extremely significant.

## 3 Results and Discussion

### 3.1 Validation

The obtained LC‐method was capable of adequately separating all 22 analytes. Details on sensitivity values and calibration ranges can be obtained from Table S1. Analysis of six drug‐free human hair samples showed no interference signals at the corresponding retention times of the analytes. Calibration measurements in the low concentration range demonstrated high sensitivity of the method with signals exceeding a signal‐to‐noise (S/N) ratio of 3:1 (determining the LOD), at analyte concentrations reaching from 0.03 to 1.0 pg/mg hair. In terms of the LLOQ, ratios greater than a (S/N) ratio of 10:1 were evaluated for concentrations from 0.10 to 1.0 pg/mg hair. The LLOQs for all analytes are below those specified by the SoHT and EWDTS guidelines and, in most cases, below those of similar methods published in the literature [17, 18, 19, 20]. Calibration curves showed good linearity (*R* ^2^ > 0.99) within their concentration ranges. Due to the complexity of the hair matrix, the acceptance criteria for the bias and RSD for the repeatability and the intermediate precision were adjusted to ± 30% (bias) and ≤ 30% (RSD), respectively. As shown in supporting information Table S4, the criteria were fulfilled by all analytes at all three QC concentration levels. The method showed good extraction recoveries (> 70%) for all the analytes at both low and high concentration levels. Matrix effects were acceptable (mean values within 70% and 130%) for most of the analytes and are listed in Table S5. For some analytes, ion suppression or enhancement effects were observed. However, the standard deviations remained within acceptable limits (≤ 30%), indicating good reproducibility. Therefore, the measurements were considered analytically acceptable.

### 3.2 Participant Characteristics and Medication History

To date, a total of 118 patients have been recruited for the study and hair samples have been successfully collected in all cases. The gender distribution of the cohort participants was 62% male (*n* = 73) and 38% female (*n* = 45), and the median age was 50 days (range: 3–5039 days). The collected hair was mostly of small sample weights (median: 1.99 mg), ranging from 0.13 to 23.2 mg, as to be expected with children and neonates. All hair colors from light blonde to black were represented in the cohort, with most of the hair samples being light brown. In most cases, the medication regimen included multiple opioids. As detailed in Table 1, the three most commonly administered opioids were fentanyl (*n* = 104), sufentanil (*n* = 89), and morphine (*n* = 114).

**TABLE 1:** Administered opioids and cumulative doses stated in micrograms per kilogram bodyweight (μg/kgBW).

Opioids	*N* cases involving treatment	Cumulative medication dose (μg/kgBW)		
		Median	Mean	Range
Fentanyl	104	26.8	186.4	1.0–6140
Sufentanil	89	14.5	19.9	0.17–150
Remifentanil	20	35.3	44.5	5.9–118
Alfenanil	2	31.5	31.5	9.0–54.0
Morphine	114	3350	7000	18.0–90,000
Hydromorphone	3	2800	4960	2025–10,060
Methadone	14	177	1190	64.0–10,600

Fentanyl was commonly employed perioperatively, has a potency that is 50–100 times higher than morphine, and was administered for a median duration of 3 days. This resulted in markedly lower cumulative doses (median: 26.8 μg/kgBW) compared to morphine. Similar potency differences apply to sufentanil (500–1000 times morphine) and remifentanil (200 times morphine) [1], both primarily used intraoperatively. This led to minimal cumulative doses of 14.5 μg/kgBW for sufentanil and 35.3 μg/kgBW for remifentanil (both medians). In contrast to the other fentalogs, remifentanil has an extremely short plasma half‐life (a few minutes) due to rapid ester hydrolysis [1]. Morphine, on the other hand, was predominantly used for postoperative pain management. Medication was usually continued over a prolonged period of time (median: 5 days), often leading to tolerance build‐up and the need for tapering to avoid withdrawal symptoms. Additionally, higher doses are generally required to achieve therapeutic efficacy with morphine, leading to substantial cumulative doses (median: 3350 μg/kgBW). The other opioids (hydromorphone and methadone) were employed for opioid rotation therapy when prolonged pain management was necessary, in cases of intolerance or as an adjunct for perioperative pain management in visceral surgery. For interpretation of the results below, it is important to note that hydromorphone is both a metabolite of morphine and a drug administered independently for analgesia in this study.

### 3.3 Authentic Hair Samples

Positivity rates and measured analyte concentrations in hair (median, mean and range) are presented in Table 2. For an analyte to be verified as detected, a signal greater than the respective LOD had to be recorded. All types of medically administered opioids as well as most targeted metabolites were repeatedly detected in the cohort's hair. As discussed in detail below, most of these analytes have never been detected in the hair of children and neonates before.

**TABLE 2:** Positivity rates and obtained analyte concentrations from the authentic hair samples.

	Positivity rate in hair	*N* cases c > LLOQ	Hair concentration (pg/mg)		
			Median	Mean	Range
Opioids					
Fentanyl	103/104 (99%)	99	4.03	14.8	0.10–343
Sufentanil	59/89 (66%)	52	0.31	0.83	0.10–8.64
Remifentanil	4/20 (20%)	4	0.14	0.20	0.10–0.43
Alfenanil	2/2 (100%)	2	0.21	0.21	0.19–0.22
Morphine	112/114 (98%)	110	27.4	130	1.0–1873
Hydromorphone	3/3 (100%)	3	5.56	8.30	3.1–16.4
Methadone	14/14 (100%)	14	46.5	729	6.0–4990
Metabolites					
Norfentanyl	41/104 (39%)	38	1.01	4.77	0.21–123
β‐Hydroxyfentanyl	55/104 (53%)	53	1.03	16.3	0.11–559
4‐ANPP	16/104 (15%)	8	0.71	1.04	0.12–2.25
Norsufentanil	32/89 (36%)	30	0.28	0.51	0.10–2.43
Remifentanil‐acid	0/20 (0%)	0	N.A.	N.A.	N.A.
Hydromorphone*	95/114 (83%)	95	5.43	18.1	0.38–244

*Note:* Hydromorphone is listed both as a therapeutic opioid and as a metabolite from morphine (marked with an asterisk). Positivity rates are based on values above the LOD, while concentration values reflect measurements with concentrations [c] above the LLOQ and within the calibration range.

Abbreviation: N.A. = not applicable.

In most cases, the opioid and metabolite concentrations measured in hair were low. Concentration values are discussed in detail below and, where possible, compared with existing literature and forensic or abstinence control cases from the Center for Forensic Hair Analysis. This in‐house data originated from adults with a mostly unknown quantity, duration, and route of exposure to the respective substances.

### 3.4 Fentanyl

Out of 104 patients with documented fentanyl administration, the analyte was detected in the hair of 103 patients. The measured concentrations ranged from 0.10 to 343 pg/mg, with a median of 4.03 pg/mg hair. Comparative values for fentanyl concentrations in hair of children and neonates could not be found in the literature, especially not those following application in a clinical and monitored setting. However, for adults, two cases could be identified where fentanyl was administered transdermally (25 μg/h) for 22 [21] and 25 consecutive days [22]. Assuming a body weight of approximately 70 kg, these doses correspond to cumulative applications of 189 and 214 μg/kgBW, respectively. These values fall within the range of doses administered in the present study (1.0–6140 μg/kgBW). The hair concentrations found in the two cases in the hair segments corresponding to the drug application period were 480 pg/mg and 100 pg/mg hair, respectively. These values are notably higher than most of those observed in the present study. However, due to the limited number of comparative cases (*n* = 2), further conclusions regarding comparability cannot be drawn. In‐house statistics from 132 fentanyl‐positive cases, analyzed over the past 2 years (2022–2024), revealed hair concentrations ranging from 1 to 2200 pg/mg (median: 10.0 pg/mg; mean: 160 pg/mg). Here, too, the concentrations were generally higher than those found in children and neonates. Due to the undisclosed nature and quantity of fentanyl consumed in the in‐house cases, the resulting data are only comparable to a limited extent.

### 3.5 Fentalogs

As illustrated in Table 2, the reported analyte concentrations of the fentanyl analogs (sufentanil, remifentanil, and alfentanil) were often near the limit of quantification. In certain cases, analyte concentrations were presumably below the sensitivity limit of the method, which would account for instances where no findings were obtained despite documented opioid administration. Comparative literature hair concentration data for children and neonates is currently unavailable and remains scarce even for adults. Only for sufentanil has such concentration data been found: a tumor patient received a cumulative dose of 7750 μg sufentanil (approximately 111 μg/kgBW) over 5 days, resulting in a hair concentration of 10 pg/mg as determined by GC–MS/MS [23]. Despite higher medication doses (up to 150 μg/kgBW) being administered in the present study, the highest observed value in hair was 8.64 pg/mg. In a forensic case reported by Kintz et al. [24], hair analysis of a suspected anesthesiologist involving unknown amounts of consumed sufentanil revealed similarly low concentrations (2 pg/mg hair), aligning with the low concentration range observed in our study. Additionally, two cases involving alfentanil detection were presented [24]. One case concerned another anesthesiologist abusing alfentanil who was reported to have a hair concentration of 30 pg/mg. The other case involved a postmortem examination of a 46‐year‐old nurse, which revealed an alfentanil hair concentration of 2 pg/mg. The concentrations measured in these cases were again higher than in the two cases examined in our study (0.22 and 0.19 pg/mg).

The positivity rate for remifentanil was 20%, with 4 cases out of 20 where remifentanil was detected in hair. Despite being administered at higher doses compared to fentanyl, its detectability and measured concentrations were notably low. This is likely attributable to remifentanil's extremely short plasma half‐life, which is only a few minutes, as stated above. The incorporation of substances into hair via the bloodstream supplying the dermal hair papilla is a key pathway for deposition [10]. Due to the brief plasma residence time of remifentanil, only a minimal amount of the substance can be incorporated into hair through this mechanism. To the best of our knowledge, this is the first study to report the detection of remifentanil in hair. Consequently, comparison with previously published data is not possible. The hydrolytically formed main metabolite remifentanil‐acid was the only one of the metabolites examined that could not be detected in any of the hair samples. This lack of detection could be attributed to either insufficient sensitivity of the analytical method or the acidic nature of the metabolite, which is generally known to impede incorporation into hair [10].

### 3.6 Morphine

Morphine, the most frequently administered opioid in this study, was detected in 112 out of 114 cases. The measured concentrations were significantly higher (median: 27.4 pg/mg) compared to the synthetic opioids described earlier. This was anticipated, as morphine was administered at comparatively higher doses due to its lower potency. Literature data on morphine concentrations in hair following monitored medical administration could not be identified for either children or adults. However, literature exists on morphine concentrations in children's hair in forensic contexts, though without precise information regarding the extent, type, or duration of exposure. In a study by Franz et al. [25], hair samples from children (*n* = 9) aged 1 to 13 years, whose caregivers were identified as drug users, were analyzed for various substances, including morphine. The concentrations found ranged from 10 to 30 pg/mg (median: 10 pg/mg) and were comparable with the concentrations determined in the present study. Comparing the present data to that of our in‐house case statistics (*n* = 248), involving the consumption of unknown amounts of medical morphine products (MST, Kapanol or Sevre‐long), the morphine ranges in adult hair were approximately 100 times higher (median: 2650 pg/mg hair). The medical morphine products mentioned above are primarily used in the context of opioid maintenance therapies. Therefore, among the consumers of the in‐house cohort, a relatively high level of opioid tolerance can be assumed, which typically results from prolonged use of high opioid doses. This represents a potential explanatory factor for the elevated morphine concentrations observed in the hair. Additionally, differences in drug incorporation between children and adults may also have contributed to the marked differences in hair concentrations.

### 3.7 Hydromorphone

Hydromorphone was administered as a therapeutic agent in three cases. However, in all three cases, morphine was also part of the medication regimen. This limits the ability to evaluate the extent to which hydromorphone was incorporated into hair as a result of direct administration versus its formation as a metabolite of morphine. Moreover, the observed hair concentrations (3.1–16.4 pg/mg) in these cases fell within the range previously observed for hydromorphone incorporation as a morphine metabolite (0.38–244 pg/mg). To date, no comparative data on hydromorphone concentrations in hair following its therapeutic or recreational use have been documented in the literature.

### 3.8 Methadone

Methadone was detected and quantified in all cases involving methadone treatment (*n* = 14) and showed the highest hair concentrations (median: 46.5 pg/mg) among all the quantified opioids. This result stands in contrast to the cumulative administered doses, which for methadone were nearly 20 times lower than those for the comparatively higher‐dosed morphine. These findings suggest that methadone is relatively well incorporated into the cohort's hair. Literature on hair concentration ranges of methadone in children or neonates following monitored administration is unavailable. However, forensic case data for children exposed to methadone are relatively abundant. In a review by Wang et al. [26], 12 cases were reported of children aged 1 to 14 years who were likely exposed to methadone, with hair concentrations ranging from 50 to 21,000 pg/mg (median: 440 pg/mg). Even though the exact exposure and duration were unknown, the concentrations observed in these cases were relatively high compared to those of other opioids in children's hair. This trend is also reflected in our in‐house statistics on methadone. In cases from 2020 to 2024 (*n* = 910), we observed values ranging from 2 to 84,000 pg/mg (median: 5600), which are significantly higher than the values found for morphine. A possible explanation for the apparently efficient incorporation of methadone into hair is its basic and lipophilic nature, which is known to promote the incorporation of substances into hair [10].

As outlined in the introduction, children's hair is particularly susceptible to external contamination. The vast majority of the cohort included in this study consisted of neonates who had never left the hospital environment. Therefore, significant external contamination of the authentic hair samples—for example, from smoke or airborne particles—was considered unlikely. However, if this analytical method is to be applied in the future to hair samples from children living in a different environment (e.g. drug‐using parents), the possibility of external contamination must be carefully considered. If the hair samples are of sufficient length and amount, a decontamination step prior to analysis, as described in the protocol of Scholz et al. [12] should be considered.

### 3.9 Quantitative Relationship Between Dose and Hair Concentration

**FIGURE 1:**
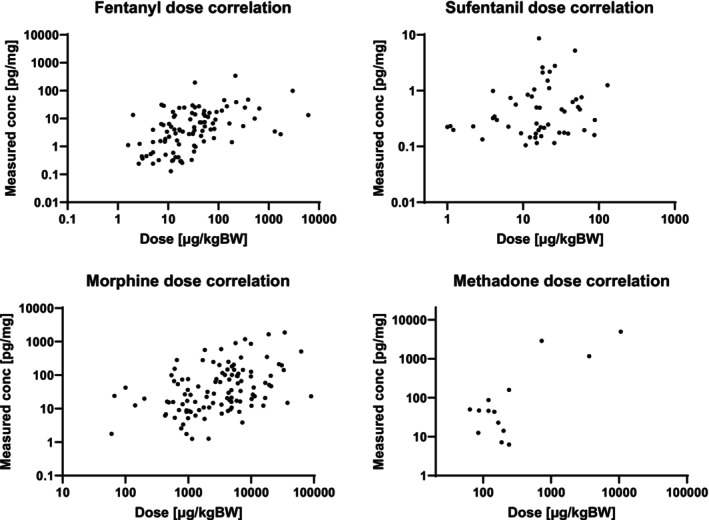
Medical doses of fentanyl, sufentanil, morphine, and methadone plotted against concentrations in hair.

For fentanyl, sufentanil, morphine, and methadone, correlations between administered opioid doses and corresponding hair concentrations were assessed and visualized by plotting the respective data points (see Figure 1). For the remaining opioids (remifentanil, alfentanil, and hydromorphone), the number of data points was more limited and insufficient for statistical analysis. A significant, medium‐strong, positive correlation (Spearman) between dose and hair concentration could be determined for fentanyl (*r* = 0.512, *p* < 0.0001) and morphine (*r* = 0.410, *p* < 0.0001). For sufentanil (*r* = 0.191, *p* = 0.175) and methadone (*r* = 0.375, *p* = 0.187), a positive trend was observed, though it did not reach statistical significance. As stated in the introduction, distinguishing between single and chronic exposure of an individual to a drug is challenging in hair analysis, especially with hair of children or neonates, due to the differences in their hair structure compared to adults. However, our findings suggest that at least for fentanyl and morphine, hair analysis results in children and neonates can provide valuable supplemental information regarding the underlying extent of exposure to the drug.

### 3.10 Metabolite‐to‐Drug Ratio in Hair

Based on the above‐listed concentration values of the metabolites of fentanyl (norfentanyl, β‐hydroxyfentanyl, and 4‐ANPP), sufentanil (norsufentanil), and morphine (hydromorphone), the ratios to their parent drug concentrations were calculated (see Table 3). To our knowledge, none of these metabolites has ever been detected in pediatric hair following controlled drug administration. Furthermore, the metabolite norsufentanil has never been quantified in hair in general. These values will serve as a reliable reference source for the interpretation of hair analysis results in the future, particularly for the differentiation between active substance intake and contamination.

**TABLE 3:** Concentration ratios of the respective metabolites to the opioids fentanyl, sufentanil, and morphine in authentic hair samples.

Metabolite/parent analyte	Ratio		
	Median	Mean	Range
Norfentanyl/fentanyl	0.10	0.15	0.016–0.788
β‐Hydroxyfentanyl/fentanyl	0.09	0.12	0.007–0.423
4‐ANPP/fentanyl	0.01	0.13	0.0003–0.613
Norsufentanil/sufentanil	0.28	0.51	0.10–2.43
Hydromorphone/morphine	0.11	0.11	0.058–0.230

The norfentanyl/fentanyl ratio is used routinely by many laboratories, including ours, as an indicator of active fentanyl use. Based on our in‐house data from 244 cases from the years 2018 to 2024, a median ratio of 0.17 was calculated, which is slightly higher than the ratio calculated in the present study (0.10).

Among all fentanyl metabolites, β‐hydroxyfentanyl was detected most frequently and at the highest concentrations. Spearman's correlation revealed a significantly stronger relationship between the administered dose and the concentration of β‐hydroxyfentanyl (*r* = 0.776, *p* < 0.0001) compared to norfentanyl (*r* = 0.490, *p* = 0.0024). These findings suggest that the metabolite ratio of β‐hydroxyfentanyl may be more suitable for interpreting fentanyl hair analysis results, particularly in young populations.

The analyte 4‐ANPP is both a metabolite of fentanyl and a commonly observed impurity in illicitly produced fentanyl and its analogs, in which it is used as a synthesis precursor [27, 28, 29]. As such, it is currently under discussion as a potential marker for the consumption of illicitly manufactured fentanyl or fentalogs. However, the extent to which 4‐ANPP is incorporated into hair when formed purely through metabolic processes has remained unclear. In studies involving users of illicit fentanyl, such as the study by Salomone et al. [30], significantly higher 4‐ANPP ratios (*n* = 146, median: 0.05, mean: 0.23) were observed. In contrast, the norfentanyl/fentanyl ratio in the same study (*n* = 154, median: 0.08, mean: 0.10) was similar to the ratios observed in the present study. Since both metabolites are formed via the same metabolic pathways, the significantly higher 4‐ANPP ratio is unlikely to result from physiological differences between children and adults. These findings suggest that the fentanyl analyzed in Salomone's study was likely contaminated with 4‐ANPP, which may explain the substantial shift in the 4‐ANPP ratio, thus supporting the hypothesis that 4‐ANPP may serve as a reliable marker for the consumption of illicitly produced fentanyl.

Norsufentanil was detected in hair at relatively high concentrations compared with its parent drug (sufentanil), resulting in a relatively high metabolite ratio (median: 0.28). As outlined earlier, to the best of our knowledge, norsufentanil has previously never been detected in hair. The metabolite ratio determined in this study, following the monitored administration of the parent drug, thus represents a valuable parameter for interpreting respective forensic cases.

The measured concentrations for hydromorphone as a metabolite of morphine (cases in which hydromorphone was applied as a drug were excluded) showed a high Spearman's correlation (*r* = 0.979, *p* < 0.0001) and resulted in a median ratio of 0.11. This ratio has never been determined in hair origin from children and neonates before. As shown by the work of Madry et al., for adults the ratio has already proved to be a valuable parameter for the interpretation of cases involving active exposure and contact to morphine or heroin [31]. In‐house data on morphine and hydromorphone from 2015 to 2025 (*n* = 781) showed a median ratio of 0.11 in adult hair—aligning with the value observed in the present study.

## 4 Conclusion

Our validated hair analysis method proved successful in detecting and quantifying opioids and their metabolites in the hair of children and neonates. Fentanyl, its analogs (remifentanil, sufentanil, and alfentanil), as well as key metabolites (norfentanyl, β‐hydroxyfentanyl, 4‐ANPP, and norsufentanil), were detected for the first time in this type of cohort. Notably, remifentanil and norsufentanil had never been detected before in hair in general, irrespective of age. The quantified opioid concentrations were notably below comparative values from adult hair and were often near the method's quantification limits. This underscores the necessity for highly sensitive analytical methods when dealing with potent medications in pediatric hair. The clinical nature of the study, with comprehensive access to patient and medication data, ensures high reliability and makes our findings a valuable resource for future interpretation of opioid hair analysis results in children and neonates. The data demonstrate significant correlations between administered opioid doses and measured hair concentrations. This finding suggests that, despite structural differences in hair of young populations, hair analysis can provide valuable supportive information for the differentiation between acute and chronic opioid intake. Furthermore, the determined metabolite‐to‐parent drug ratios will be instrumental in differentiating active intake from environmental contact or exposure—an issue of particular relevance in cases involving children or neonates living in environments where drug use is prevalent. In the future, attempts will be made to recruit more patients in order to increase the sample size with a resulting increase in statistical power. This will eventually enable further research questions and hypotheses to be answered regarding patient and medication‐related covariates.

## Supporting information


**Table S1:** Analytes with their assigned group and internal standard for quantification, calibration ranges, regression type and obtained limit of detection and lower limit of quantification (LOD and LLOQ).
**Table S2:** Calibrator and QC samples with their respective analyte concentration in hair. Each sample was spiked with 50 μL IS solution (40 pg/μL) resulting in a final hair concentration of 100 pg/mg for the deuterated analytes.
**Table S3:** Analyte retention times (RT), ion transitions and optimized compound specific source parameters, including the entrance potential (EP), collision energy (CE) and cell exit potential (CXP) for the positive ionization with electron spray.
**Table S4:** Bias, repeatability and precision values obtained for the quantifier ion of the analytes of the LC–MS/MS method. RSD_R_ = relative standard deviation of repeatability, RSD_T_ = relative standard deviation of time‐different intermediate precision.
**Table S5:** Matrix effects and recoveries in % obtained for the quantifier ion of the analytes of the LC–MS/MS method. Recovery rates were calculated based on normalized signal areas, using the respective internal standards.

## Data Availability

The data that support the findings of this study are available from the corresponding author upon reasonable request.
