# Supplementary material for: Evaluation of Therapeutic Opioids in Hair of Neonatal and Pediatric Patients
Source: Drug Test Anal. 2025 Jul 27;17(11):2246–54. doi: 10.1002/dta.3935 (PMC12580163; doi:10.1002/dta.3935)
Supplement: Supplementary file 1 — Table S1: Analytes with their assigned group and internal standard for quantification, calibration ranges, regression type and obtained limit of detection and lower limit of quantification (LOD and LLOQ). Table S2: Calibrator and QC samples with their respective analyte concentration in hair. Each sample was spiked with 50 μL IS solution (40 pg/μL) resulting in a final hair concentration of 100 pg/mg for the deuterated analytes. Table S3: Analyte retention times (RT), ion transitions and optimized compound specific source parameters, including the entrance potential (EP), collision energy (CE) and cell exit potential (CXP) for the positive ionization with electron spray. Table S4: Bias, repeatability and precision values obtained for the quantifier ion of the analytes of the LC–MS/MS method. RSDR = relative standard deviation of repeatability, RSDT = relative standard deviation of time‐different intermediate precision. Table S5: Matrix effects and recoveries in % obtained for the quantifier ion of the analytes of the LC–MS/MS method. Recovery rates were calculated based on normalized signal areas, using the respective internal standards. [file DTA-17-2246-s001.docx]

**Supplemental**

Table S1: Analytes with their assigned group and internal standard for quantification, calibration ranges, regression type and obtained limit of detection and lower limit of quantification (LOD and LLOQ).

| **Analyte / Group** | **Internal standard (IS)** | **Calibration range**  **[pg/mg hair]** | **Regression** | **LOD**  **[pg/mg hair]** | **LLOQ [pg/mg hair]** | **Linearity (R^2^)** |
| --- | --- | --- | --- | --- | --- | --- |
| 4-ANPP / 1 | Fentanyl-d_5_ | 0.1 - 500 | Wagner | 0.05 | 0.1 | 0.9985 |
| Acetylcodeine / 2 | Morphine-d_3_ | 1.0 - 5000 | Wagner | 0.30 | 1.0 | 0.9944 |
| 6-Monoacetylmorphine / 2 | Morphine-d_3_ | 1.0 - 5000 | Wagner | 0.30 | 1.0 | 0.9951 |
| Alfentanil / 1 | Fentanyl-d_5_ | 0.1 - 500 | Wagner | 0.03 | 0.1 | 0.9950 |
| β-Hydroxyfentanyl / 1 | Fentanyl-d_5_ | 0.1 - 500 | Wagner | 0.05 | 0.1 | 0.9953 |
| Codeine / 2 | Morphine-d_3_ | 1.0 - 5000 | Wagner | 0.30 | 1.0 | 0.9933 |
| Dihydrocodeine / 2 | Morphine-d_3_ | 1.0 - 5000 | Wagner | 0.30 | 1.0 | 0.9924 |
| Fentanyl / 1 | Fentanyl-d_5_ | 0.1 - 500 | Wagner | 0.03 | 0.1 | 0.9963 |
| Hydrocodone / 1 | Morphine-d_3_ | 0.1 - 500 | Wagner | 0.05 | 0.1 | 0.9981 |
| Hydromorphone / 1 | Morphine-d_3_ | 1.0 - 5000 | Wagner | 0.05 | 0.1 | 0.9960 |
| Methadone / 2 | Methadone-d_9_ | 1.0 - 5000 | Wagner | 1.00 | 1.0 | 0.9976 |
| Morphine / 2 | Morphine-d_3_ | 1.0 - 5000 | Wagner | 0.10 | 1.0 | 0.9985 |
| Naloxone / 2 | Fentanyl-d_5_ | 0.1 - 500 | Wagner | 0.50 | 1.0 | 0.9996 |
| Norfentanyl / 1 | Norfentanyl-d_5_ | 0.1 - 500 | Wagner | 0.03 | 0.1 | 0.9985 |
| Norsufentanil / 1 | Norsufentanil-d_3_ | 0.1 - 500 | Wagner | 0.03 | 0.1 | 0.9914 |
| Oxycodone / 2 | Morphine-d_3_ | 1.0 - 5000 | Wagner | 0.30 | 1.0 | 0.9974 |
| Oxymorphone / 2 | Morphine-d_3_ | 1.0 - 5000 | Wagner | 0.30 | 1.0 | 0.9988 |
| Pethidine / 1 | Morphine-d_3_ | 0.1 - 500 | Wagner | 0.03 | 0.1 | 0.9984 |
| Remifentanil / 1 | Fentanyl-d_5_ | 0.1 - 500 | Wagner | 0.05 | 0.1 | 0.9976 |
| Remifentanil-acid / 1 | Fentanyl-d_5_ | 0.1 - 500 | Wagner | 0.10 | 0.1 | 0.9953 |
| Sufentanil / 1 | Fentanyl-d_5_ | 0.1 - 500 | Wagner | 0.05 | 0.1 | 0.9974 |
| Tramadol / 2 | Morphine-d_3_ | 1.0 - 5000 | Wagner | 1.00 | 1.0 | 0.9867 |

Table S2: Calibrator and QC samples with their respective analyte concentration in hair. Each sample was spiked with 50 µL IS solution (40 pg/µL) resulting in a final hair concentration of 100 pg/mg for the deuterated analytes.

| **Group** | **Analyte concentration in hair [pg/mg hair]** | | | | | | | | | | |
| --- | --- | --- | --- | --- | --- | --- | --- | --- | --- | --- | --- |
|  | **Cal. 1** | **Cal. 2** | **Cal. 3** | **Cal. 4** | **Cal. 5** | **Cal. 6** | **Cal. 7** | **Cal. 8** | **QC**_low_ | **QC**_med_ | **QC**_high_ |
| 1 | 0.1 | 1 | 5 | 10 | 50 | 100 | 300 | 500 | 0.12 | 100 | 400 |
| 2 | 1 | 5 | 50 | 100 | 500 | 1000 | 2000 | 5000 | 1.2 | 500 | 4000 |

Table S3: Analyte retention times (RT), ion transitions and optimized compound specific source parameters, including the entrance potential (EP), collision energy (CE) and cell exit potential (CXP) for the positive ionization with electron spray.

| **Substance** | **Q1 mass [Da]** | **Q3 mass [Da]** | **RT [min]** | **EP [V]** | **CE [V]** | **CXP [V]** |
| --- | --- | --- | --- | --- | --- | --- |
| 4-ANPP | 281.1 | 188.1/105.0 | 5.88 | 10 | 23/39 | 10/12 |
| Acetylcodeine | 342.1 | 152.0/165.1 | 4.58 | 10 | 91/59 | 12/12 |
| 6-Monoacetylmorphine | 328.0 | 164.9/211.0 | 3.56 | 10 | 51/35 | 14/16 |
| Alfentanil | 417.2 | 268.1/197.1 | 5.59 | 10 | 23/35 | 14/10 |
| β-Hydroxyfentanyl | 353.2 | 204.0/146.0 | 5.34 | 10 | 30/33 | 13/10 |
| Codeine | 300.0 | 215.2/165.1 | 3.12 | 10 | 33/53 | 20/16 |
| Dihydrocodeine | 302.1 | 201.2/171.1 | 3.08 | 10 | 37/55 | 18/10 |
| Fentanyl | 337.2 | 188.1/105.0 | 5.84 | 10 | 32/53 | 13/12 |
| Hydrocodone | 300.1 | 199.1/128.1 | 3.66 | 10 | 41/75 | 16/12 |
| Hydromorphone | 286.1 | 185.0/157.1 | 1.22 | 10 | 39/55 | 16/14 |
| Methadone | 310.1 | 76.9/223.1 | 6.77 | 10 | 81/27 | 10/18 |
| Morphine | 286.1 | 152.1/128.1 | 1.23 | 10 | 83/77 | 18/14 |
| Naloxone | 328.1 | 212.1/310.2 | 3.18 | 10 | 51/27 | 24/20 |
| Norfentanyl | 233.1 | 84.1/177.1 | 3.94 | 10 | 23/21 | 10/22 |
| Norsufentanil | 277.1 | 128.0/96.0 | 4.74 | 10 | 19/27 | 06/12 |
| Oxycodone | 316.1 | 212.2/187.1 | 3.45 | 10 | 55/35 | 18/14 |
| Oxymorphone | 302.0 | 198.0/284.1 | 1.56 | 10 | 59/27 | 16/18 |
| Pethidine | 248.1 | 220.1/174.1 | 4.78 | 10 | 29/29 | 20/10 |
| Remifentanil | 377.1 | 228.1/317.0 | 4.90 | 10 | 27/21 | 12/16 |
| Remifentanil-acid | 363.0 | 214.0/303.0 | 4.46 | 10 | 27/21 | 12/18 |
| Sufentanil | 387.1 | 238.0/355.0 | 6.53 | 10 | 27/25 | 12/18 |
| Tramadol | 264.1 | 58.0/42.0 | 4.45 | 10 | 49/103 | 10/6 |
| Morphine-d_3_ | 289.1 | 201.0 | 1.23 | 10 | 37 | 13 |
| Norsufentanil-d_3_ | 280.1 | 131.0 | 4.73 | 10 | 19 | 8 |
| Fentanyl-d_5_ | 342.2 | 137.2 | 5.82 | 10 | 45 | 8 |
| Norfentanyl-d_5_ | 238.2 | 182.1 | 3.94 | 10 | 23 | 12 |
| Methadone-d_9_ | 319.3 | 268.1 | 6.77 | 10 | 23 | 16 |

Table S4: Bias, repeatability and precision values obtained for the quantifier ion of the analytes of the LC-MS/MS method. RSD_R_ = relative standard deviation of repeatability, RSD_T_ = relative standard deviation of time-different intermediate precision.

| **Substance** | **QC Level** | **Theoretical conc.**  **[pg/20mg hair]** | **Calculated conc.**  **[pg/20mg hair]** | **Bias, %** | **RSD_R_, %** | **RSD_T_, %** |
| --- | --- | --- | --- | --- | --- | --- |
| 4-ANPP | low | 2.4 | 2.6 | 8.0 | 12.4 | 14.1 |
|  | med | 2000 | 2358.3 | 17.9 | 2.5 | 14.5 |
|  | high | 8000 | 7769.2 | -2.9 | 6.8 | 9.7 |
| Acetylcodeine | low | 24 | 26.2 | 9.1 | 10.4 | 13.1 |
|  | med | 10000 | 9480.0 | -5.2 | 5.6 | 11.3 |
|  | high | 80000 | 70582.1 | -11.8 | 8.2 | 17.5 |
| 6-Monoacetylmorphine | low | 24 | 25.7 | 7.3 | 11.8 | 11.5 |
|  | med | 10000 | 10401.4 | 4.0 | 7.7 | 6.7 |
|  | high | 80000 | 76224.7 | -4.7 | 8.4 | 14.2 |
| Alfenanil | low | 2.4 | 2.6 | 8.8 | 4.7 | 8.2 |
|  | med | 2000 | 2238.0 | 11.9 | 6.4 | 12.4 |
|  | high | 8000 | 7863.3 | -1.7 | 6.0 | 10.1 |
| β-Hydroxyfentanyl | low | 2.4 | 2.9 | 19.0 | 7.5 | 18.7 |
|  | med | 2000 | 2090.8 | 4.5 | 3.0 | 11.5 |
|  | high | 8000 | 8170.5 | 2.1 | 5.3 | 11.4 |
| Codeine | low | 24 | 26.5 | 10.3 | 11.2 | 14.2 |
|  | med | 10000 | 9296.9 | -7.0 | 7.8 | 14.3 |
|  | high | 80000 | 72527.2 | -9.3 | 8.1 | 18.0 |
| Dihydrocodeine | low | 24 | 26.7 | 11.2 | 7.5 | 11.4 |
|  | med | 10000 | 9550.4 | -4.5 | 6.6 | 14.0 |
|  | high | 80000 | 70535.7 | -11.8 | 9.7 | 16.8 |
| Fentanyl | low | 2.4 | 2.8 | 14.7 | 0.7 | 7.2 |
|  | med | 2000 | 2339.5 | 17.0 | 4.3 | 7.8 |
|  | high | 8000 | 9002.9 | 12.5 | 2.0 | 8.9 |
| Hydrocodone | low | 2.4 | 2.8 | 18.6 | 24.9 | 22.0 |
|  | med | 2000 | 2023.4 | 1.2 | 5.1 | 7.5 |
|  | high | 8000 | 8020.8 | 0.3 | 6.5 | 7.3 |
| Hydromorphone | low | 2.4 | 2.7 | 12.9 | 11.3 | 13.6 |
|  | med | 2000 | 1421 | -28.9 | 3.7 | 3.0 |
|  | high | 8000 | 8256.1 | 3.2 | 9.8 | 13.9 |
| Methadone | low | 24 | 28.6 | 19.0 | 4.7 | 15.4 |
|  | med | 10000 | 11606.3 | 16.1 | 5.1 | 7.3 |
|  | high | 80000 | 71609.5 | -10.5 | 6.7 | 11.9 |
| Morphine | low | 24 | 25.3 | 5.6 | 5.9 | 9.3 |
|  | med | 10000 | 9765.2 | -2.4 | 6.8 | 10.1 |
|  | high | 80000 | 76825.2 | -4.0 | 10.1 | 13.7 |
| Naloxone | low | 24 | 25.8 | 7.6 | 8.9 | 12.2 |
|  | med | 10000 | 9753.7 | -2.5 | 8.3 | 8.0 |
|  | high | 80000 | 78981.0 | -1.3 | 9.3 | 15.9 |
| Norfentanyl | low | 2.4 | 2.6 | 9.6 | 15.4 | 12.5 |
|  | med | 2000 | 2198.0 | 9.9 | 3.7 | 8.0 |
|  | high | 8000 | 7923.9 | -1.0 | 1.7 | 5.0 |
| Norsufentanil | low | 2.4 | 2.6 | 2.6 | 9.9 | 11.0 |
|  | med | 2000 | 2273.9 | 13.7 | 4.0 | 7.4 |
|  | high | 8000 | 7790.7 | -2.6 | 4.5 | 6.7 |
| Oxycodone | low | 24 | 27.0 | 12.5 | 10.7 | 11.3 |
|  | med | 10000 | 9151.6 | -8.5 | 7.2 | 13.7 |
|  | high | 80000 | 72423.2 | -9.5 | 7.7 | 19.5 |
| Oxymorphone | low | 24 | 25.3 | 5.2 | 6.8 | 6.0 |
|  | med | 10000 | 10216.0 | 2.2 | 7.7 | 8.6 |
|  | high | 80000 | 77924.7 | -2.6 | 7.8 | 15.0 |
| Pethidine | low | 2.4 | 2.7 | 12.2 | 9.6 | 12.3 |
|  | med | 2000 | 1981.3 | -0.9 | 4.1 | 12.5 |
|  | high | 8000 | 7633.9 | -4.6 | 6.0 | 9.8 |
| Remifentanil | low | 2.4 | 2.5 | 4.9 | 8.1 | 13.6 |
|  | med | 2000 | 2128.0 | 6.4 | 7.2 | 14.7 |
|  | high | 8000 | 7945.5 | -0.7 | 5.4 | 9.3 |
| Remifentanil-acid | low | 2.4 | 2.6 | 9.8 | 11.2 | 9.2 |
|  | med | 2000 | 2151.7 | 7.6 | 5.4 | 11.9 |
|  | high | 8000 | 7792.9 | -2.6 | 10.2 | 14.6 |
| Sufentanil | low | 2.4 | 2.7 | 11.2 | 8.4 | 12.2 |
|  | med | 2000 | 2153.3 | 7.7 | 10.6 | 12.5 |
|  | high | 8000 | 8014.2 | 0.2 | 5.5 | 10.7 |
| Tramadol | low | 24 | 25.3 | 5.5 | 9.9 | 14.5 |
|  | med | 10000 | 9167.8 | -8.3 | 9.8 | 14.2 |
|  | high | 80000 | 70426.5 | -12.0 | 10.7 | 17.4 |

Table S5: Matrix effects and recoveries in % obtained for the quantifier ion of the analytes of the LC-MS/MS method. Recovery rates were calculated based on normalized signal areas, using the respective internal standards.

| **Substance** | **QC Sample** | **Theoretical conc.**  **[pg/20mg hair]** | **Recovery** | | **Matrix effect** | |
| --- | --- | --- | --- | --- | --- | --- |
|  |  |  | **Mean [%]** | **SD [%]** | **Mean [%]** | **SD [%]** |
| 4-ANPP | low | 2.4 | 96.5 | 24.4 | 83.5 | 15.8 |
|  | high | 8000 | 93.6 | 6.9 | 88.0 | 14.2 |
| Acetylcodeine | low | 24 | 98.3 | 16.3 | 100.0 | 3.2 |
|  | high | 80000 | 92.7 | 5.8 | 100.8 | 7.1 |
| 6-Monoacetylmorphine | low | 24 | 75.8 | 10.2 | 96.6 | 10.2 |
|  | high | 80000 | 98.4 | 3.6 | 90.0 | 6.2 |
| Alfentanil | low | 2.4 | 92.0 | 6.0 | 86.7 | 9.9 |
|  | high | 8000 | 96.2 | 7.7 | 96.5 | 7.4 |
| β-Hydroxyfentanyl | low | 2.4 | 87.2 | 8.1 | 87.2 | 8.8 |
|  | high | 8000 | 94.3 | 10.1 | 82.7 | 7.8 |
| Codeine | low | 24 | 94.4 | 7.2 | 108.6 | 10.2 |
|  | high | 80000 | 86.5 | 8.5 | 108.5 | 6.7 |
| Dihydrocodeine | low | 24 | 99.2 | 8.8 | 85.1 | 11.7 |
|  | high | 16000 | 95.9 | 5.6 | 93.6 | 5.6 |
| Fentanyl | low | 2.4 | 88.5 | 11.5 | 57.3 | 14.9 |
|  | high | 8000 | 97.0 | 15.0 | 67.2 | 15.0 |
| Hydrocodone | low | 2.4 | 96.6 | 18.3 | 109.8 | 13.2 |
|  | high | 8000 | 88.3 | 4.5 | 89.6 | 5.8 |
| Hydromorphone | low | 2.4 | 75.8 | 10.2 | 120.6 | 14.7 |
|  | high | 8000 | 98.4 | 3.6 | 98.5 | 4.4 |
| Methadone | low | 24 | 96.5 | 9.8 | 91.3 | 3.8 |
|  | high | 80000 | 98.1 | 7.2 | 88.4 | 10.2 |
| Morphine | low | 24 | 92.8 | 9.2 | 87.2 | 15.7 |
|  | high | 80000 | 90.0 | 4.5 | 85.0 | 4.8 |
| Naloxone | low | 24 | 97.7 | 6.1 | 83.0 | 7.1 |
|  | high | 80000 | 93.1 | 4.9 | 106.0 | 5.4 |
| Norfentanyl | low | 2.4 | 96.2 | 10.6 | 80.1 | 7.1 |
|  | high | 8000 | 92.2 | 4.5 | 92.8 | 10.6 |
| Norsufentanil | low | 2.4 | 99.7 | 12.8 | 86.6 | 8.3 |
|  | high | 8000 | 88.3 | 5.4 | 91.6 | 10.6 |
| Oxycodone | low | 24 | 95.4 | 11.1 | 94.9 | 10.1 |
|  | high | 80000 | 94.3 | 5.8 | 100.2 | 4.8 |
| Oxymorphone | low | 24 | 92.3 | 5.3 | 87.2 | 6.5 |
|  | high | 80000 | 99.9 | 6.1 | 87.5 | 4.1 |
| Pethidine | low | 2.4 | 99.9 | 6.7 | 85.4 | 6.3 |
|  | high | 8000 | 94.5 | 5.5 | 88.0 | 10.0 |
| Remifentanil | low | 2.4 | 96.0 | 8.0 | 107.5 | 13.8 |
|  | high | 8000 | 94.5 | 4.7 | 97.0 | 9.8 |
| Remifentanil-acid | low | 2.4 | 96.4 | 13.8 | 162.2 | 21.8 |
|  | high | 8000 | 95.9 | 6.6 | 126.5 | 4.8 |
| Sufentanil | low | 2.4 | 98.8 | 5.4 | 77.3 | 14.3 |
|  | high | 8000 | 96.6 | 9.8 | 82.7 | 11.8 |
| Tramadol | low | 24 | 93.4 | 8.9 | 104.3 | 19.0 |
|  | high | 80000 | 91.9 | 6.4 | 88.7 | 11.9 |
